# Gating defects of disease-causing *de novo* mutations in Ca_v_1.3 Ca^2+^ channels

**DOI:** 10.1080/19336950.2018.1546518

**Published:** 2018-11-22

**Authors:** Alexandra Pinggera, Giulia Negro, Petronel Tuluc, Morris J. Brown, Andreas Lieb, Jörg Striessnig

**Affiliations:** a Department of Pharmacology and Toxicology, Institute of Pharmacy and Center for Molecular Biosciences, University of Innsbruck, Innsbruck, Austria; b William Harvey Research Institute, Queen Mary University of London, London, UK

**Keywords:** Aldosterone producing adenomas, autism spectrum disorder, Ca^2+^ channel, CACNA1D, mutation

## Abstract

Recently, we and others identified somatic and germline *de novo* gain-of-function mutations in *CACNA1D*, the gene encoding the α1-subunit of voltage-gated Ca_v_1.3 Ca^2+^-channels. While somatic mutations identified in aldosterone producing adenomas (APAs) underlie treatment-resistant hypertension, germline *CACNA1D* mutations are associated with a neurodevelopmental disorder characterized by a wide symptomatic spectrum, including autism spectrum disorder. The number of newly identified *CACNA1D* missense mutations is constantly growing, but their pathogenic potential is difficult to predict *in silico*, making functional studies indispensable to assess their contribution to disease risk.

Here we report the functional characterization of previously identified *CACNA1D* APA mutations F747L and M1354I using whole-cell patch-clamp electrophysiology upon recombinant expression in tsA-201 cells. We also investigated if alternative splicing of Ca_v_1.3 affects the aberrant gating of the previously characterized APA mutation R990H and two mutations associated with autism spectrum disorder (A479G and G407R). Splice-variant dependent gating changes are of particular interest for germline mutations, since the relative expression of Ca_v_1.3 splice variants differs across different tissues and within brain regions and might therefore result in tissue-specific phenotypes. Our data revealed a complex gain-of-function phenotype for APA mutation F747L confirming its pathogenic role. Furthermore, we found splice-variant dependent gating changes in R990H, A749G and G407R. M1354I did not change channel function of Ca_v_1.3 splice variants and should therefore be considered a rare non-pathogenic variant until further proof for its pathogenicity is obtained. Our new findings together with previously published data allow classification of pathogenic *CACNA1D* mutations into four categories based on prototypical functional changes.

## Introduction

Different classes of voltage-gated Ca^2+^ channels serve a central role for key physiological processes by generating inward Ca^2+^ currents in response to changes in membrane potentials in electrically excitable cells [1–3]. Ca^2+^ influx through these channels drives not only membrane depolarization but, through Ca^2+^ ions as universal second messenger, also regulates a large variety of intracellular Ca^2+^ dependent processes. These include muscle contraction, gene transcription, hormone secretion, neurotransmitter release, synaptic excitability and plasticity [1–3]. Their subcellular targeting as well as the dynamics of Ca^2+^ entry need to be tightly controlled by accessory subunits, post-translational modification, lipid- and protein interactions, as well as alternative splicing [1,2]. The occurrence of human channelopathies resulting from even minor changes in Ca^2+^ channel activity [4–8] further emphasizes the importance of strictly regulated channel gating and targeting for normal physiological function.

We [9–12] and others [13–16] have recently described *de novo* missense mutations in *CACNA1D* encoding the pore-forming α1-subunit of Ca_v_1.3 L-type channels. These included somatic mutations identified in aldosterone-producing adenomas [APAs, 11,12,15], as well as germline mutations from patients with neurodevelopmental disorders characterized by autism spectrum disorder (ASD) and/or intellectual disability with or without seizures [9,10,13–15]. Three of these patients further displayed primary aldosteronism or hyperinsulinism [13,15]. All so far functionally characterized mutations – both from germline and somatic mutations – induced pronounced gating changes, evident either as robust shifts in activation and inactivation voltage to more negative potentials and/or strong reductions of *I_Ca_* inactivation, thus permitting enhanced Ca^2+^ entry through the channel. This suggests that Ca_v_1.3 gain-of-function cannot only drive excessive aldosterone production in APAs [Ca^2+^ is the critical steroidogenic second messenger in zona glomerulosa cells, 17] but might also confer high risk for neuropsychiatric and neurological disorders [18].

Due to the known physiological role of Ca_v_1.3 for brain development and function [2,18] and the recent findings of recurrent *de novo* germline *CACNA1D* mutations in patients with neuropsychiatric and neurological symptoms this gene has been incorporated in custom genetic panels for clinical diagnosis, including autism/intellectual disability (Autism/ID Xpanded Panel).

Based on findings in Ca_v_1.3-deficient mice [19] and humans [20] heterozygous *de novo* loss-of-function mutations are expected to be clinically silent and only missense mutations with gating changes permitting enhanced Ca^2+^ signaling are likely to be disease relevant. However, the functional consequences of such mutations are difficult to predict *in silico*. Therefore, newly identified rare missense variants have to be functionally characterized in heterologous expression systems to confirm their disease causing potential. So far this has been accomplished for seven patients with germline *CACNA1D* mutations [six different mutations, 9,10,13–15] and six somatic mutations occurring in APAs [11,12,15,21]. Interestingly, several of the functionally verified germline mutations causing a neurodevelopmental disorder are also found in APAs [I750M, G403D, V401L, 18]. Therefore, if a germline mutation in patients with a neurodevelopmental syndrome is also reported in APAs this would be strong support for its pathogenic role and guide genetic and clinical diagnosis. The number of *CACNA1D* mutations in APAs in precursor lesions (aldosterone producing cell clusters, APCCs,) is constantly growing [>40, 18,22,23]. All these mutations are highly likely to allow enhanced Ca^2+^ signaling though Ca_v_1.3 channels because they are selected by their ability to drive Ca^2+^ -dependent aldosterone production. However, despite this selection pressure not all predicted high confidence *CACNA1D* APA and APCC mutations may strongly contribute to disease risk because other, yet unknown mechanisms could be responsible. Notably, a substantial fraction of excessive aldosterone production in APAs is due to mechanisms not explained by somatic variants in known genes [24]. This emphasizes the need for further functional characterization of *CACNA1D* APA mutations to verify their causative roles.

Here we report the analysis of three APA mutations (F747L, R990H, M1354I) which we have previously reported together with other C*ACNA1D* mutations in a cohort of 152 subjects [11]. We also investigated if alternative splicing of Ca_v_1.3 α1 can affect mutational effects on channel function in R990H, M1354I and in two previously reported ASD mutations A749G and G407R. Indeed, we found splice-variant dependent gating changes in R990H, A749G and G407R. Our data confirm a gain-of function phenotype also for F747L but not for M1354I. Finally, our experiments support our previous hypothesis that an ω-current through mutant R990H rather than altered gating explains its pathogenic potential.

## Experimental procedures

### Complementary DNA constructs

Human wild-type Ca_v_1.3α_1_-subunits (reference sequence EU363339) comprising alternative exons 8a and 42 (long C-terminal splice variant) or 43s [short C-terminal splice variant, 25] were previously cloned into the pGFP^minus^ vector (no GFP tag, CMV promoter) [25]. Mutations were introduced into Ca_v_1.3 splice variants using standard polymerase chain reaction approaches and verified by Sanger sequencing (Eurofins Genomics, Ebersberg, Germany). When co-expressed with auxiliary β_3_ and α_2_δ-1 subunits in tsA-201 cells, all mutant α_1_-subunits were detected with the expected molecular mass in Western blots and at densities comparable to wild-type (n ≥ 3 transfections for each mutant).

### Cell culture and transfection

tsA-201 cells were maintained in culture and transiently transfected with wild-type (WT) or mutant Ca_v_1.3 α_1_- and auxiliary β_3_- (NM_012828) and α_2_δ-1- (NM_001082276) subunits as previously described [9,26]. GFP was co-transfected to visualize transfected cells.

### Electrophysiological recordings in tsA-201 cells

For whole-cell patch-clamp recordings borosilicate glass electrodes (203-776-0664 Warner Instruments and 64-0792, Harvard Apparatus, USA) with a final resistance of 1.5–3.0 MΩ were pulled using a micropipette puller (Sutter instruments, P-97) and fire polished afterwards (Microforge, Narishinge MF-830). All recordings were performed at room temperature (21–23 °C) in whole-cell configuration using the Axopatch 200B amplifier (Axon instruments), digitized at 50 kHz (Digitizer 1322A, Axon instruments), low-pass filtered at 5 kHz and compensated for 60–90 % of the series resistance. The recording solutions contained in mM: *bath*: 15 CaCl_2_, 10 HEPES, 150 choline-Cl and 1 MgCl_2_, adjusted to pH 7.4 with CsOH; *intracellular*: 135 CsCl, 10 HEPES, 10 Cs-EGTA, 1 MgCl_2_, 4 Na_2_ATP adjusted to pH 7.4 with CsOH. The holding potential (HP) was set to −80 mV. To determine the current-voltage (I-V) relationship, a 30 ms (short splice variants) or 50 ms (long splice variants) square pulse protocol to different voltages was applied. Resulting I-V curves were fitted from −80 mV to + 40 mV to the following equation:

$$I=G_{max}\left(V-V_{rev}\right)/\left(1+exp\left(-\left(V-V0.5\right)/k\right)\right)$$

where V_rev_ is the extrapolated reversal potential, V the test potential, I the peak current, G_max_ the maximum conductance, V_0.5_ the half maximal activation voltage and k the slope factor. The voltage-dependence of Ca^2+^ conductance was fitted according to a Boltzman distribution:

$$G=G_{max}/\left(1+exp\left(-\left(V-V_{0.5}\right)/k\right)\right).$$

Estimates for changes in channel open probability and/or single channel conductance were obtained as described [25] by normalizing the ionic tail current to the amplitude of the integrated ON-gating current (Q_ON_) of the same pulse at the V_rev_. The Q_ON_ at the V_rev_ was also used to determine the surface expression of the channel complexes.

For analysis of current densities, surface expression and estimation of the open probability only recordings from the same transfections were compared.

Steady-state inactivation was measured by applying a control test pulse (20 ms to the voltage of maximal inward current, V_max_) followed by 5-s conditioning steps to various potentials and a subsequent 20-ms test pulse to V_max_ (30-s recovery between protocols). Inactivation was calculated as the ratio between the current amplitudes of the test versus control pulse. Steady-state inactivation parameters were obtained by fitting the data to a modified Boltzmann equation:

$$G=\left(1-G_{max}\right)/\left(1+exp\left(\left(V-V_{0.5}\right)/k\right)\right)+G_{max.}$$

To investigate inactivation kinetics, cells were depolarized for 5 s to the V_max_, normalized (I/I_max_) and the remaining current (R) at different time points was determined. Recovery from inactivation was determined by a 10 ms test pulses to V_max_ at the indicated time after a 1-s conditioning pulse to V_max_. Differences in the inactivation after repeated stimuli were determined after applying 100-ms square pulses to V_max_ at a frequency of 3 Hz. Holding potential between the steps was −60 mV.

Leak subtraction was performed either offline (steady-state inactivation, 5-s inactivation, recovery from inactivation and pharmacological experiments) or online using P/4 protocol. Recordings were junction potential corrected by −9.3 mV for Ca^2+^ and by −8.6 for Ba^2+^ as charge carrier, as previously described [27].

### Statistics

Data were analyzed using Clampfit 10.2 (Axon Instruments), Microsoft Excel, SigmaPlot 8 (Systat Software, Inc) and GraphPad Prism 5 software (GraphPad software, Inc). All values are presented as mean ± S.E.M. for the indicated number of experiments (*n*) unless stated otherwise. Data were analyzed by unpaired Student’s t-test, Mann-Whitney test, and one-way ANOVA followed by Bonferroni post-hoc test. Statistical significance was set at *p < 0*.05.

## Results

### Splice-variant dependent effects of ASD mutations G407R and A749G

**Figure 1.**
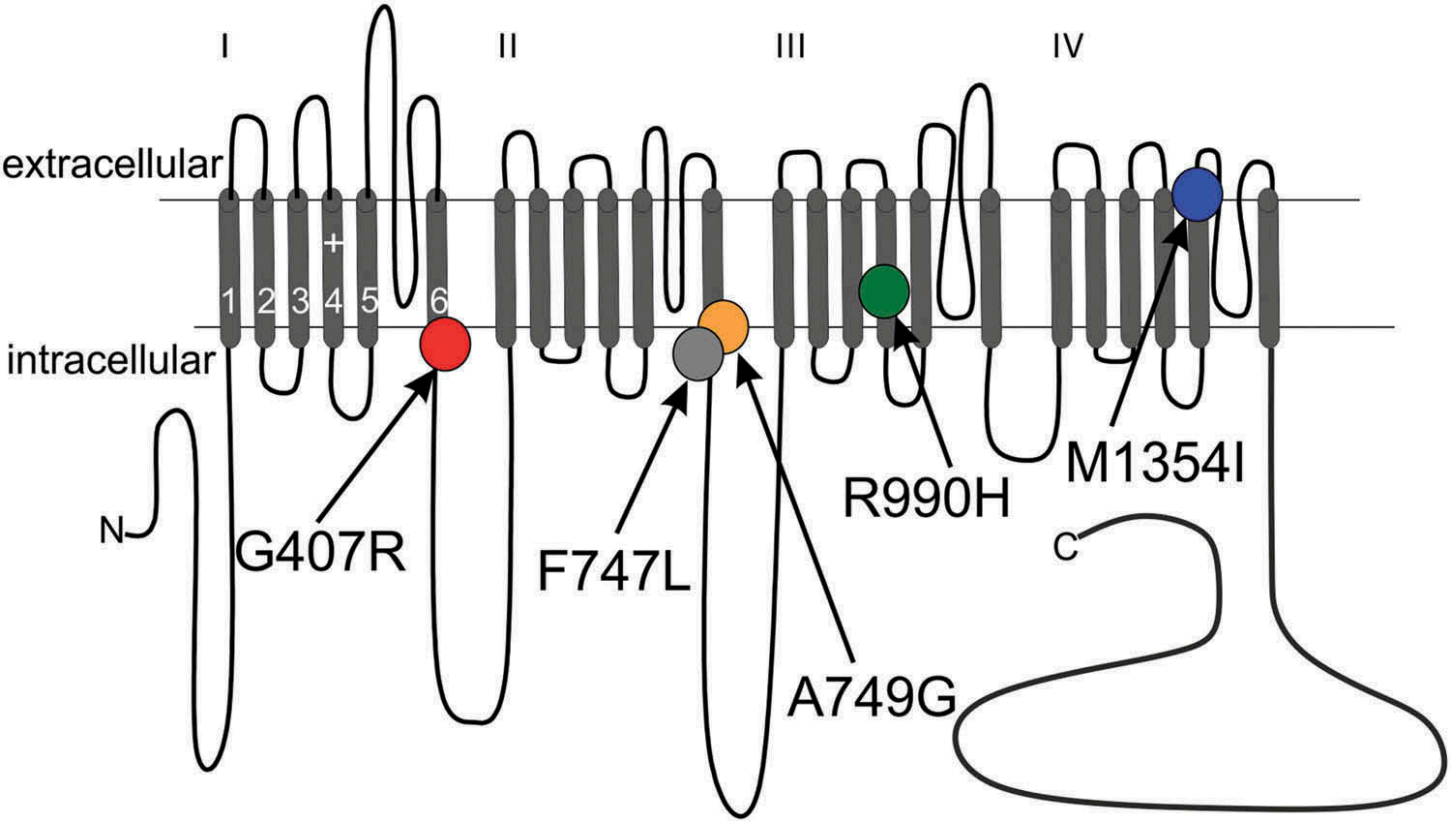
Transmembrane topology of the Ca_v_1.3 α1-subunit. The pore-forming subunit of voltage gated Ca^2+^ channels consist of four transmembrane repeats each comprising six membrane spanning helices connected by intra- and extracellular linkers. Segments (S) 1–4 of each repeat comprise the voltage- sensing domain, whereas segments 5–6 line the pore. The positions of missense mutations identified in ASD (G407R, A749G) and APAs (F747L, R990H, M1354I) are indicated. Mutations G407R (IS6), F747L (IIS6) and A749G (IIS6) reside within the so-called activation gate at the distal part of the S6 segment. Mutation R990H affects the third of the five gating charges within the IIIS4 helix of the voltage sensing domain and M1354I is localized at the distal part of segment IVS5 facing the extracellular side. For references see text.

We have recently shown that two *de novo* missense mutations, G407R and A749G (Figure 1), found in two patients with ASD cause severe gating changes that are expected to result in aberrant Ca_v_1.3-mediated Ca^2+^ signaling in the brain. Together with three other mutations (V401L, G407D, I750M) that cause an even more severe neurodevelopmental syndrome [9,15], this strongly suggests that these mutations confer a high risk for these diseases [18]. Ca_v_1.3 also undergoes extensive alternative splicing, giving rise to a major C-terminal long splice variant (Ca_v_1.3_L_) and a number of C-terminal short splice variants, with Ca_v_1.3_43s_ (Ca_v_1.3_S_) being the most abundant one in the brain [25,28–30]. C-terminal splicing removes a modulatory domain allowing channels to undergo major changes in voltage-and Ca^2+^-dependent gating resulting in activation at lower voltages, enhanced Ca^2+^-dependent inactivation and increased channel open probability [25,27,28,31]. We have previously reported gating effects of the germline mutations A749G and G407R (situated at the intracellular end of IS6 and IIS6, respectively) only in the human C-terminally long (Ca_v_1.3_L_) isoform [10]. To test for potential splice-variant dependent effects we now introduced both mutations into human Ca_v_1.3_S_ to compare their functional consequences with those in Ca_v_1.3_L_ under identical experimental conditions.

**Figure 2.**
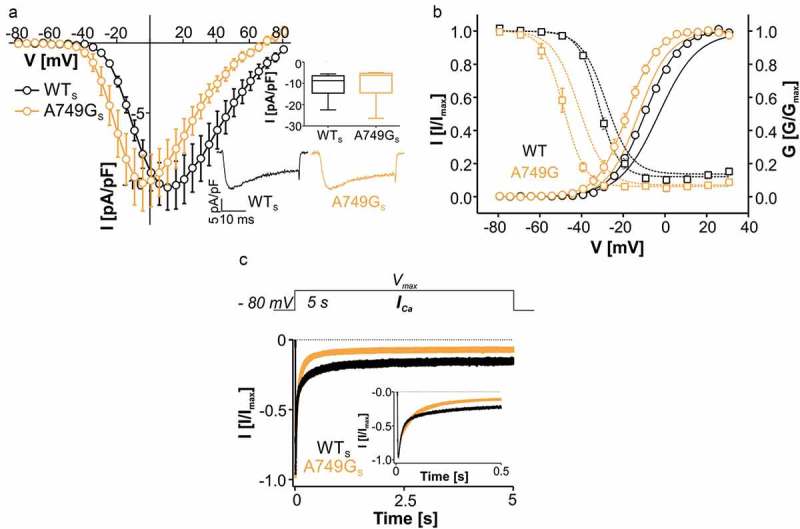
Biophysical properties of Ca_v_1.3 mutant A749G_S._ (a) Current-voltage relationships (*I_Ca_*, mean ± S.E.M.; 50-ms depolarizations to indicated voltages) of WT_S_ and A749G_S_. Only WT data recorded in parallel on the same days (> 3- different transfections) were included. Inset (upper): Box plots of WT_S_ and A749G_S_ peak current densities. In contrast to the long splice variant, no significant difference was observed in the current amplitudes (median, 5/95 percentile, [pA/pF]): WT_S_: −8.74 (−22.4/-5.54) n = 10; A749G_S_: −6.18 (−26.4/-4.94), n = 12; p = 0.339, Mann Whitney test). Inset (lower): Representative *I_Ca_* traces from WT_S_ and A749G_S_ during depolarizations to V_max_. (b) Steady-state activation (circles, solid lines) and inactivation (squares, dashed lines) of WT_S_ (black symbols) and A749G_S_ (orange symbols). For statistics and n-numbers see Table 1. Lines without symbols represent the corresponding voltage-dependence of activation and inactivation for full length WT_L_ and A749G_L_ recorded under identical conditions (taken from reference 10). (c) Inactivation time course of WT_S_ (n = 35) and A749G_S_ (n = 9) during depolarization to V_max_ for 5 s. Normalized traces are shown as mean ± S.E.M. Inactivation was best described by a bi-exponential decay. The faster inactivation of the mutant resulted from a slightly larger contribution of the fast inactivating component (WT_S_: 79.1 ± 1.11 %, n = 26, A749G_S_: 84.3 ± 2.21 %, p = 0.036, n = 8) and faster inactivation of the slow component (WT_S_: τ_slow_ = 602 ± 21.8 ms, A749G_S_: 420 ± 59.6 ms, p = 0.0012), despite a moderately slower fast inactivation (WT_S_: τ_fast_ = 36.5 ± 1.8 ms, A749G_S_: 57.9 ± 7.99 ms, p = 0.0004). Additionally, inactivation was more complete in the mutant (remaining current: WT_S_: 14.6 ± 2.5 %, A749G_S_: 5.65 ± 1.19 %, p = 0.061, for statistics see Table 2). Statistical significance has been determined by unpaired Student’s t-test.

Mutation A749G_S_ resulted in a similarly strong negative shift of the voltage dependence of activation and steady-state inactivation as the long splice variant (Figure 2(a,b), Table 1; historical data for long splice variants measured under identical conditions are given for comparison). Since wild-type Ca_v_1.3_S_ activates and inactivates at more negative voltages than Ca_v_1.3_L_ [Figure 2(a,b), Table 1, 26], a substantial fraction of A749G_S_ inactivated already at negative voltages unable to activate channels (closed-state inactivation), as evident from the steady-state activation and inactivation curves (Figure 2(b)). At −50 mV, where channels were not activated, only 1.13 ± 0.01 % and 2.51 ± 0.01 % of WT_L_ and WT_S_ channels are inactivated, whereas for A749G_L_ 19.2 ± 0.03 % and for A749G_S_ even 41.8 ± 0.07 % were already unavailable (p < 0.0001 for A749G_L_ *vs*. WT_L_, A749G_S_ *vs*. WT_S_ and A749G_L_ vs. A749G_S_, one-way ANOVA followed by Bonferroni post hoc test). Since our measurements require recordings with 15 mM Ca^2+^ as a charge carrier, voltage-dependent activation and inactivation curves would be shifted by about −18 mV at physiological Ca^2+^ concentrations [2 mM Ca^2+^; 25]. Therefore, about half of the A749G_S_ channels are predicted to be already inactivated at membrane potentials close to resting potentials. In the heterozygous individuals this would decrease the availability of A749Gs in comparison to wild-type channels in neurons with more positive membrane potentials, such as for example substantia nigra and ventral tegmental area dopamine neurons. This reveals both, gain- and loss of function properties of these mutations depending not only on the activity patterns of different neurons but also strongly affected by the relative abundance of long and short splice variants.

**Table 1.** Activation and inactivation parameters for ASD mutations A749G and G407R.

	activation				inactivation			
	V_rev_ [mV]	Slope [mV]	V_0.5_ [mV]	n	V_0.5_ [mV]	Slope [mV]	Remaining current [%]	n
WT_S_	64.9 ± 1.04	7.71 ± 0.11	−9.49 ± 0.42	66	−31.7 ± 0.88	4.31 ± 0.12	11.5 ± 2.18	25
AG_S_	54.7 ± 1.46***	6.61 ± 0.11***	−18.3 ± 1.34***	13	−48.6 ± 1.58***	4.27 ± 0.16	6.01 ± 1.50	9
GR_S_	61.6 ± 1.77	7.68 ± 0.28	−15.3 ± 2.20***	14	nd			
*WT_L_*	*67.7 ± 1.14*	*8.92 ± 0.20*	*−2.55 ± 1.05*	*29*	*25.7 ± 2.08*	*5.56 ± 0.23*	*14.4 ± 3.12*	*18*
*AG_L_*	*60.3 ± 0.83^###^*	*7.14 ± 0.20^###^*	*−12.3 ± 0.87^###^*	*27*	*−41.1 ± 1.07^###^*	*5.82 ± 0.18*	*9.43 ± 1.49*	*14*
*GR_L_*	*54.7 ± 2.32^###^*	*7.90 ± 0.30*	*−6.58 ± 1.41*	*13*	*nd*			

All values are presented as mean ± S.E.M. Number of independent transfections >3. Parameters were obtained after fitting normalized (I/I_max_) I-V relationships or normalized (I/I_max_) steady-state inactivation curves, as described in Methods. Statistical analysis was performed using one-way ANOVA and Bonferroni post-hoc test (activation) or unpaired Student’s t-test (steady-state inactivation). Significance **^, ##^ p < 0.01, ***^, ###^ p < 0.001 in comparison to WT_S_ (*) or WT_L_ (^#^) (statistical analysis has been performed separately for long and short splice variants). n, number of experiments; nd, not determined; V_0.5_, half maximal activation/inactivation voltage; V_rev_, reversal potential; WT, wild-type. Note small differences among WT recordings within different sets of experiments (Tables 1, 3 and 5) because WT control experiments were always carried out in parallel with mutated channels. Reference data, which were already published previously, are indicated in italics (taken from reference 10).

**Table 2.** Remaining current upon 5-s depolarization for ASD mutations A749G and G407R.

	R50 [%]	R500 [%]	R5000 [%]	n
WT_S_	41.1 ± 1.84	20.7 ± 2.05	13.5 ± 2.09	35
AG_S_	46.2 ± 3.92	11.8 ± 1.87	8.16 ± 1.84	9
GR_S_	73.6 ± 4.52***	62.6 ± 5.39***	59.9 ± 5.62***	8
*WT_L_*	*69.5 ± 3.2*	*22.8 ± 2.42*	*6.12 ± 1.13*	*15*
*AG_L_*	*60.0 ± 4.31*	*7.22 ± 0.76^###^*	*3.12 ± 0.56^###^*	*6*
*GR_L_*	*99.2 ± 0.65^###^*	*98.1 ± 1.33^###^*	*80.2 ± 3.52^###^*	*13*

All values are presented as mean ± S.E.M. Number of independent transfections ≥2. *I_Ca_* inactivation normalized to I_max_ was obtained 50, 500 and 5000 ms after depolarization to V_max_. Statistical analysis was performed using one-way ANOVA and Bonferroni post-hoc test. Significance ***^, ###^ p < 0.001 in comparison to WT_S_ (*) or WT_L_ (^#^). n, number of experiments; R, remaining current; WT, wild-type. Reference data, which were already published previously, are indicated in italics (taken from reference 10).

**Table 3.** Activation and inactivation parameters for APA mutations F747L and M1354I.

	activation				inactivation			
	V_rev_ [mV]	Slope [mV]	V_0.5_ [mV]	n	V_0.5_ [mV]	Slope [mV]	Remaining current [%]	n
WT_L_	70.4 ± 0.67	9.06 ± 0.12	1.37 ± 0.60	63	−27.0 ± 0.69	5.54 ± 0.18	14.4 ± 2.02	28
FL_L_	53.8 ± 0.91***	6.62 ± 0.22***	−15.6 ± 0.71***	30	−29.3 ± 0.98*	4.45 ± 0.32**	32.2 ± 2.50***	19
MI_L_	69.5 ± 0.69	9.01 ± 0.13	2.48 ± 0.79	40	−25.0 ± 0.82	5.57 ± 0.22	19.6 ± 2.26	25
WT_S_	64.9 ± 1.04	7.71 ± 0.11	−9.49 ± 0.42	66	−31.7 ± 0.88	4.31 ± 0.12	11.5 ± 2.18	25
MI_S_	62.5 ± 1.36	7.36 ± 0.18	−9.01 ± 0.64	19	−30.8 ± 0.86	3.92 ± 0.18	13.8 ± 2.49	13

All values are presented as mean ± S.E.M. Number of independent transfections >3. Parameters were obtained after fitting normalized (I/I_max_) I-V relationships or normalized (I/I_max_) steady-state inactivation curves, as described in Methods. Statistical analysis was performed by one-way ANOVA and Bonferroni post-hoc test (long splice variants) or unpaired Student’s t-test (short splice variants). Significance * p < 0.05, ** p < 0.01, *** p < 0.001 in comparison to WT_L._ n, number of experiments; V_0.5_, half maximal activation/inactivation voltage; V_rev_, reversal potential; WT, wild-type.

**Table 4.** Remaining current upon 5-s depolarization for APA mutations F747L and M1354I.

	R50 [%]	R500 [%]	R5000 [%]	n
WT_L_	71.7 ± 2.38	29.4 ± 2.11	9.83 ± 1.86	28
FL_L_	99.3 ± 0.83^###^	63.4 ± 4.78^###^	32.2 ± 3.52^###^	10
MI_L_	69.9 ± 3.64	29.7 ± 3.81	16.1 ± 3.84	12
WT_S_	41.1 ± 1.84	20.7 ± 2.05	13.5 ± 2.09	35
MI_S_	44.2 ± 3.70	23.9 ± 3.99	18.4 ± 4.34	9

All values are presented as mean ± S.E.M. Number of independent transfections >3. *I_Ca_* inactivation normalized to I_max_ was obtained 50, 500 and 5000 ms after depolarization to V_max_. Statistical analysis was performed using one-way ANOVA and Bonferroni post-hoc test (long splice variants or unpaired Student’s t-test (short splice variants). Significance ^###^ p < 0.001 in comparison to WT_L_. n, number of experiments; R, remaining current; WT, wild-type.

**Table 5.** Activation and inactivation parameters for APA mutation R990H.

	activation				inactivation			
	V_rev_ [mV]	Slope [mV]	V_0.5_ [mV]	n	V_0.5_ [mV]	Slope [mV]	Remaining current [%]	n
WT_S_	64.9 ± 1.04	7.71 ± 0.11	−9.49 ± 0.42	66	−31.7 ± 0.88	4.31 ± 0.12	11.5 ± 2.18	25
RH_S_	60.0 ± 1.73	7.55 ± 0.19	−5.37 ± 0.63***	11	−37.3 ± 0.57**	5.26 ± 0.53*	3.92 ± 2.42	8
*WT_L_*	*66.2 ± 1.10*	*9.02 ± 0.23*	*−0.65 ± 1.27*	*29*	*−30.8 ± 1.14*	*5.38 ± 0.30*	*11.3 ± 2.35*	*16*
*RH_L_*	*69.7 ± 1.57*	*8.57 ± 0.14*	*2.61 ± 0.69^#^*	*33*	*−32.5 ± 1.12*	*6.76 ± 0.39^#^*	*11.3 ± 2.05*	*18*

All values are presented as mean ± S.E.M. Number of independent transfections >3. Parameters were obtained after fitting normalized (I/I_max_) I-V relationships or normalized (I/I_max_) steady-state inactivation curves, as described in Methods. Statistical analysis was performed by unpaired Student’s t-test. Significance *^, #^ p < 0.05, **^, ##^ p < 0.01, ***^, ###^ p < 0.001 in comparison to WT_S_ (*) or WT_L_ (^#^). n, number of experiments; V_0.5_, half maximal activation/inactivation voltage; V_rev_, reversal potential; WT, wild-type. Reference data, which were already published previously, are indicated in italics (taken from reference 21).

Mutation A749G slightly accelerated inactivation kinetics of both splice variants (Table 2). Bi-exponential inactivation over a 5-s time course of A749G_S_ was faster (Figure 2), due to a significant decrease of τ_slow_ and an increase of the contribution of the fast inactivating component (Figure 2(c), see legend for statistics). τ_fast_ was slightly prolonged, which was also evident from a trend to more remaining current after 50 ms (Table 2). Although we made no attempts to separate the contribution of CDI and VDI for inactivation, the decrease of τ_slow_ must reflect a slowing of VDI by the mutation, whereas the increase in τ_fast_ suggests a slowing of CDI. This agrees with the observation of reduced CDI and enhanced VDI of this mutation in a rat Cav1.3 splice variant [16]. The authors predicted that the increase in VDI by the mutation may act in opposition to the reduced of CDI. Our data provide direct experimental support for their prediction.

As described previously [12,25] ON-gating currents (Q_ON_) were very small or absent for WT_S_ preventing measurements of the ratio of maximal tail current amplitude vs. integrated ON-gating current as an estimate for changes in open probability. Whereas A749G_L_ significantly enhanced peak current amplitudes [10] this was not observed for A749G_S_. Instead, depolarization to voltages causing maximal activation (V_max_) of WT channels resulted in smaller current amplitudes in the mutant (Figure 2(a)). This indicated reduced A749G_S_ current compared to WT, which was compensated by a higher driving force at peak current amplitudes due to its more negative activation voltage range. Since analysis of the Q_ON_ in the long splice variants revealed reduced surface expression of A749G_L_ [10], this suggests that the smaller current at WT V_max_ observed with A749G_S_ might also be due to a reduction in channel numbers at the cell surface.

**Figure 3.**
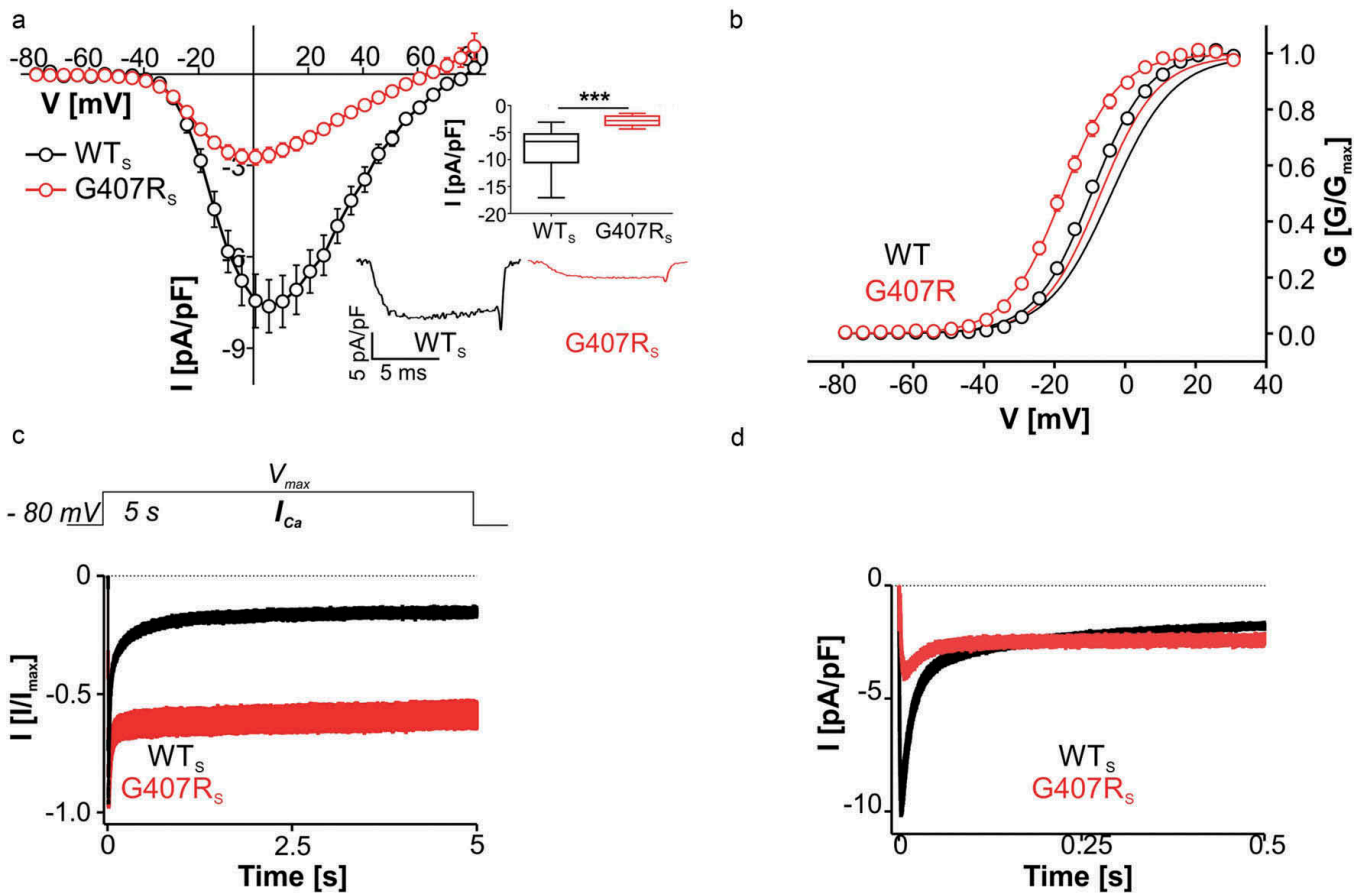
Biophysical properties of Ca_v_1.3 mutant G407R_S. _(a) Current-voltage relationships (*I_Ca_*, mean ± S.E.M.) from WT_S_ and G407R_S_ recorded in parallel on the same days (>3-different transfections). Inset (upper): Box plots showing significantly reduced peak current densities for G407R_S_ (median (5/95 percentile) [pA/pF]: WT_S_: −6.68 (−17.1/-3.09), n = 17; G407R_S_: −2.82 (−4.36/-1.46), n = 13; *** p < 0.0001, Mann Whitney test). Inset (lower): Representative *I_Ca_* traces of WT_S_ and G407R_S_ upon depolarization to V_max._ (b) Voltage dependence of WT (black circles) and G407R_S_ (red circles) activation. Lines without symbols depict the corresponding voltage-dependence of activation for full length WT_L_ (black) and G407R_L_ (red) recorded under identical conditions (taken from reference 10). Statistics for gating parameters are given in Table 1. (c) Normalized inactivation time course of *I_Ca_* after 5-s depolarization to V_max_ (mean ± S.E.M.). The fast inactivating component is reduced in G407R_S_ (n = 8) as compared to WT (n = 35) leading to slower inactivation of the mutant (for statistics see Table 2). (d) Although G407R_S_ maximal current density is significantly smaller than WT_S_, absolute G407R_S_ current is larger than WT_S_ after about 200 ms.

Splicing-dependent differences were also found for ASD mutation G407R. G407R reduced maximal current amplitudes in both C-terminally long [Table 1, 10] and short Ca_v_1.3 splice variants (Figure 3(a), Table 1). However, in contrast to G407R_L_, the voltage dependence of activation was significantly shifted in G407R_S_ to more negative voltages (Figure 3(b)). Like for G407R_L_ the inactivation time course during 5-s depolarizations was also dramatically slowed for G407R_S_ (Figure 3, Table 2). G407R_S_, in contrast to G407R_L_ [10] retained an initial fast inactivating component, most likely representing residual CDI (Figure 3(c,d)), which is more pronounced in short Ca_v_1.3 splice variants [25,31,32]. Despite reduced maximal current amplitudes, the slow inactivation resulted in larger absolute current amplitudes during prolonged depolarization than in WT_S_ channels (Figure 3(c,d)). The fraction of *I_Ca_* became larger after about 200 ms and was remarkably enhanced at the end of a 5-s pulse to V_max_ for G407R_S_ (59.9 ± 5.62 %, n = 8), compared to WT_S_ (13.5 ± 2.09 %, n = 35) (Figure 3(c), Table 2). The failure of G407R currents to inactivate prevented the measurement of steady-state inactivation parameters. Together with the more negative activation voltage range of G407R_S_, this predicts persistent inward current at even more negative (subthreshold) potentials than for G407R_L_ (Figure 3).

### APA mutations F747L and R990H – but not M1354I – alter channel gating

**Figure 4.**
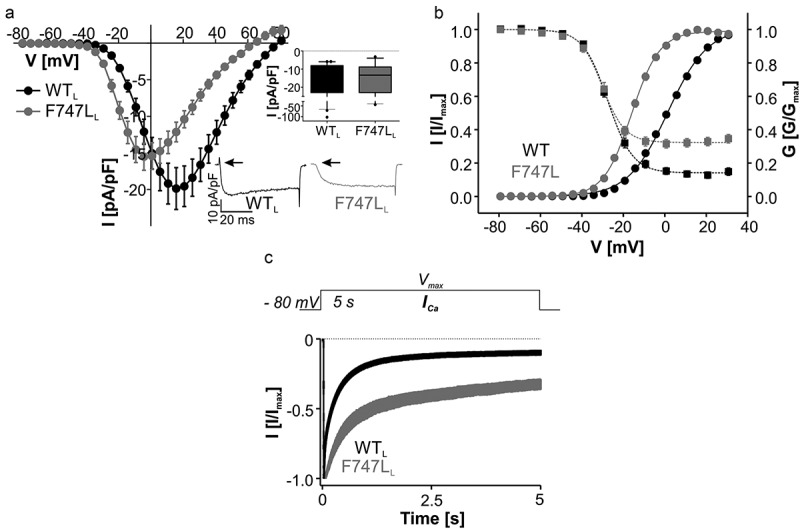
Biophysical properties of Ca_v_1.3 mutant F747L_L._ (a) Current-voltage relationships (*I_Ca_*, mean ± S.E.M.) of WT_L_ and F747L_L_ recorded in parallel on the same days (>3-different transfections). Inset (upper): Box plots of peak current densities (median, 5/95 percentile, [pA/pF]): WT_L_: −12.7 (−66.1/-5.96), n = 47; F747L_L_: −13.3 (−40.3/-3.97), n = 27). Black circles represent extreme values. Peak values were not statistically different (Mann Whitney test, p = 0.97). Inset (lower): Representative *I_Ca_* traces of WT_L_ and F747L_L_ upon depolarization to V_max_. Note the slower activation time course and the absence of Q_ON_ gating charge in F747L_L_  (arrows). (b) Steady-state activation (circles, solid lines) and inactivation (squares, dashed lines) of WT_L_ (black symbols) and F747L_L_ (gray symbols). Gating parameters and statistics are given in Table 3. (c) Normalized inactivation time course of *I_Ca_* after 5-s depolarization to V_max_ (mean ± S.E.M.) illustrating pronounced slowing of inactivation by F747_L_.(n = 10) as compared to WT_L_ (n = 28). For statistics, see Table 4.

We next determined if APA mutations F747L, R990H and M1354I (Figure 1) also interfere with proper channel function in a similar manner as described above. F747L resides at the intracellular end of IIS6 just 2 amino acids upstream of A749G. When introduced into the long splice variant F747L_L_ also strongly shifted the voltage dependence of activation (about −17 mV) to more negative potentials, while the voltage dependence of inactivation remained unaltered (Figure 4(a,b), Table 3). This dramatically broadened the voltage range in which the channel is active and increases the window current. Moreover, F747L_L_ currents inactivated more slowly and showed a significantly higher fraction of non-inactivating *I_Ca_* even after prolonged depolarizations (Figure 4(c); Table 4 for statistics). F747L_L_ also significantly slowed the time course of activation (time to peak at V_max_ [ms]: WT_L_: 6.66 ± 0.44, n = 35; F747L_L_: 30.1 ± 1.76, n = 21, p < 0.0001, unpaired Student’s t-test; traces in Figure 4(a), inset). Like for A749G_S_, current amplitudes were smaller at positive voltages suggesting overall reduced current through the mutated channel. Unfortunately, quantification of the surface expression of functional F747L_L_ was not possible because the mutation eliminated measurable Q_ON_ gating charge movement. As evident from the current traces in Figure 4(a) (inset, arrows), in 27 out of 30 recordings the Q_ON_ voltage sensor movement, which is present in WT_L_ in all cells, was not detectable. We have obtained a similar decrease in Q_ON_ relative to ionic current previously for APA mutations I750M, V259D and P1336R [11] and ASD mutation A749G [10] when introduced into the long splice variant. Taken together, F747L_L_ exhibits the typical gating changes observed for previously studied mutations clearly identifying this mutation as the cause for excess aldosterone production in APAs.

Unlike previously described APA mutations, R990H and M1354I are not located within the activation gate or the S4-S5 linkers [18,21] and may therefore induce different functional changes.

**Figure 5.**
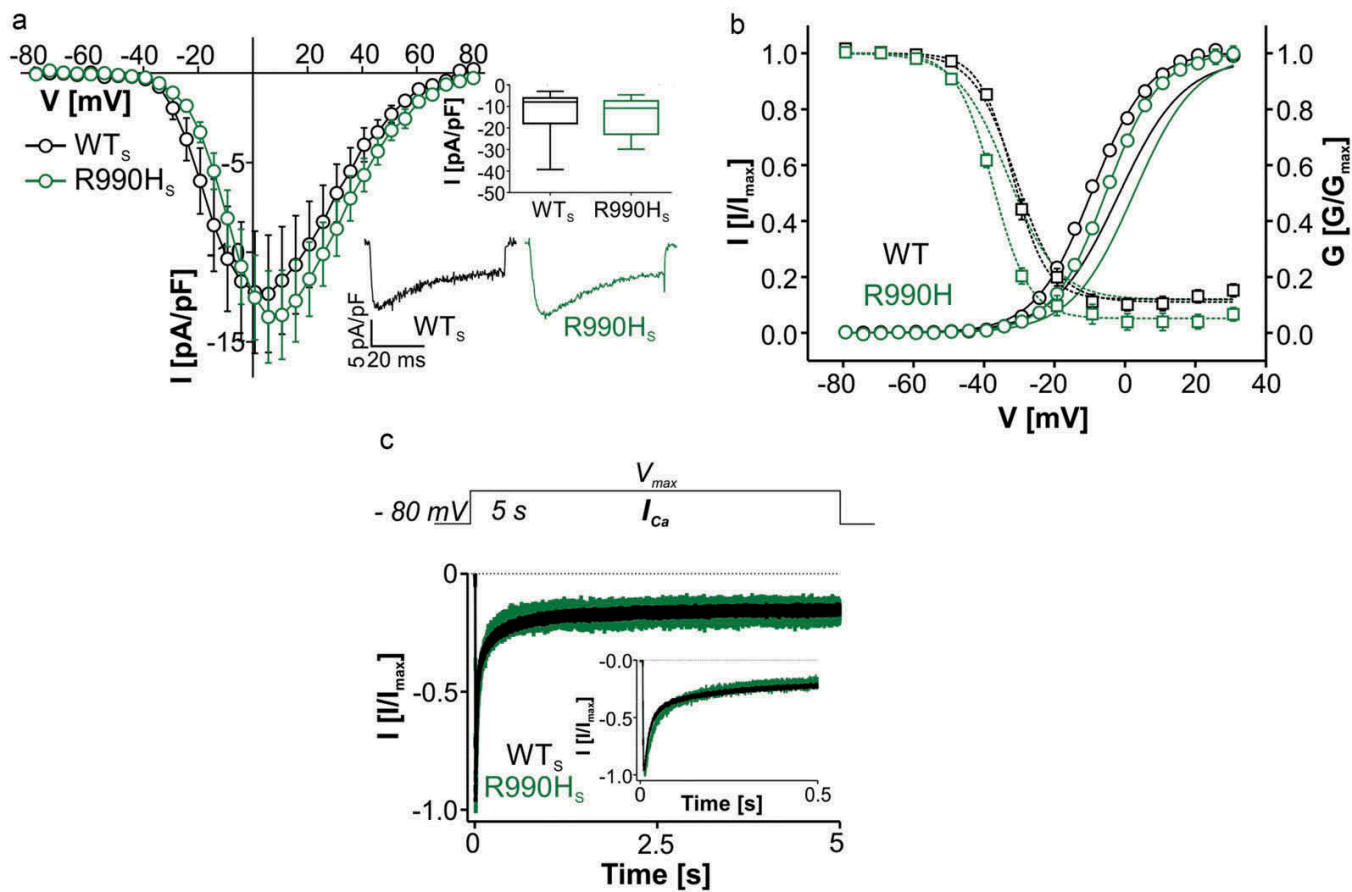
Biophysical properties of Ca_v_1.3 mutant R990H_S._ (a) Current-voltage relationships (*I_Ca_*, mean ± S.E.M.) from WT_S_ and R990H_S_ recorded in parallel on the same days (>3-different transfections). Inset (upper): Box plots of peak current densities (median (5/95 percentile, [pA/pF]): WT_S_: −7.96 (−39.2/-3.03), n = 10; R990H_S_: −10.8 (−29.8/-4.65), n = 11; p = 0.549, Mann Whitney test). Inset (lower): Representative *I_Ca_* traces of WT_S_ and R990H_S_ upon depolarization to V_max_. (b) Steady-state activation (circles, solid lines) and inactivation (squares, dashed lines) of WT_S_ and R990H_S_. For statistics and n-numbers see Table 5. Lines without symbols depict the corresponding voltage-dependence of activation and inactivation for full length WT_L_ and R990H_L_ for comparison [21] (c) Normalized inactivation time course of *I_Ca_* after 5-s depolarization to V_max_ (mean ± S.E.M.) for WT_S_ (n = 35) and R990H_S_ (n = 9). For statistics, see Table 6.

R990H replaces the third (R3) of the five S4 positive charges of the domain III voltage sensing S4 helix [21]. We have recently shown that, when introduced into the long splice variant, R990H_L_ causes gating changes compatible with a loss-of-function phenotype: it tended to reduce inward current at all voltages, and slightly but significantly shifted activation (by about 3 mV) to more positive rather than negative voltages as in all other mutations studied so far (Table 5). For this mutation we have previously provided experimental and molecular modeling evidence for an ω-current through the voltage-sensor. This can be explained by a water wire formed in the voltage-sensor of the R990H mutation, which enables cationic leak current at hyperpolarized resting membrane potentials and therefore can indirectly trigger depolarization-induced Ca^2+^ entry in APAs [21]. However, it is also possible that a gain-of-function phenotype is absent when introduced into the long but present in the short splice variant. We therefore tested this possibility by analyzing R990H_S_ (Figure 5). Like R990H_L_, R990H_S_ shifted activation voltage to more positive voltages by about 4 mV (Figure 5, Table 5). However, when introduced into the short splice variant R990H also significantly shifted steady-state inactivation by about 6 mV to more negative voltages thus even reducing window current by decreasing channel availability at negative voltages. Current density (Figure 5(a)) and the time course of inactivation (Table 6) were not affected by the mutation in neither of the splice variants. In the absence of any other significant changes observed in R990H_S_ these findings provide additional evidence for a loss-of-function phenotype of this mutation for Ca^2+^ entry through the canonical ion pore of both long and short variants of the channel. This further strengthens the view that the pathogenic gain-of-function phenotype results from its ω-current.

**Table 6.** Remaining current upon 5-s depolarization for APA mutation R990H.

	R50 [%]	R500 [%]	R5000 [%]	n
WT_S_	41.1 ± 1.84	20.7 ± 2.05	13.5 ± 2.09	35
RH_S_	46.6 ± 3.45	19.5 ± 3.73	15.1 ± 3.54	9
WT_L_	69.7 ± 3.94	24.8 ± 3.50	11.1 ± 2.94	10
RH_L_	67.1 ± 3.59	21.6 ± 3.75	13.6 ± 3.62	12

All values are presented as mean ± S.E.M. Number of independent transfections >3. *I_Ca_* inactivation normalized to I_max_ was obtained 50, 500 and 5000 ms after depolarization to V_max_. Statistical analysis was performed using unpaired Student’s t-test in comparison to WT_S_ or WT_L_. n, number of experiments; R, remaining current; WT, wild-type.

**Figure 6.**
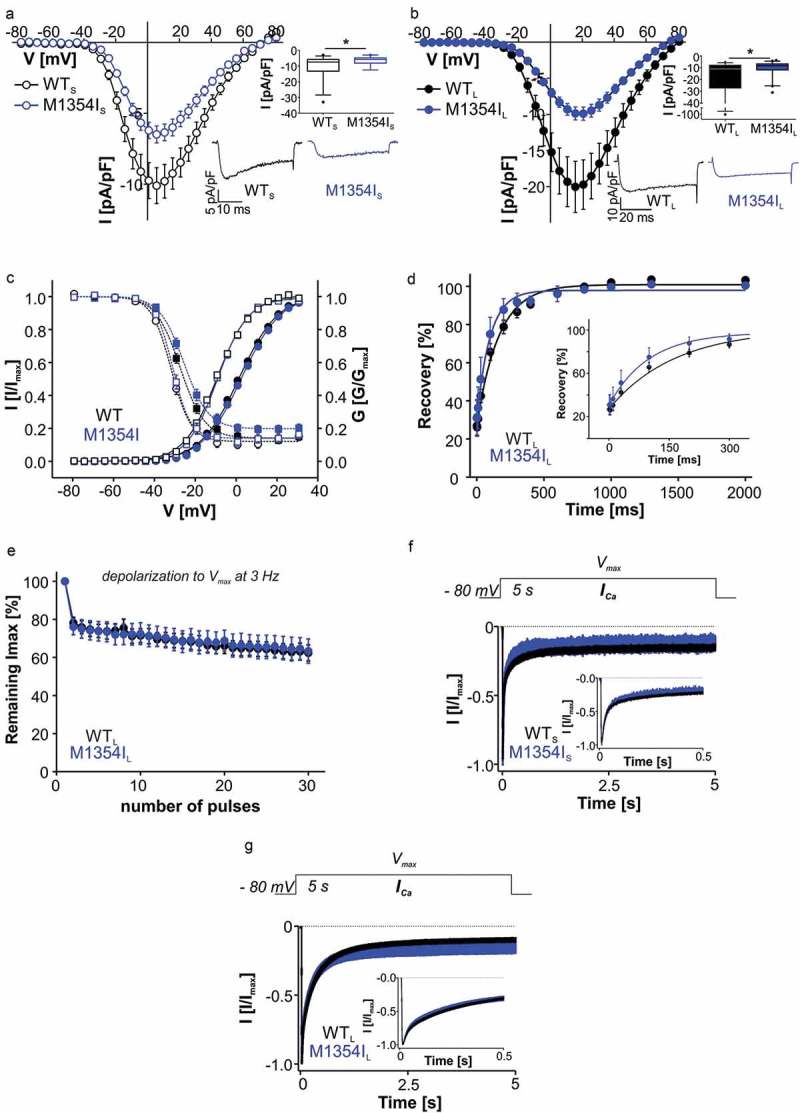
Biophysical properties of Ca_v_1.3 mutant M1354I. (a) Current-voltage relationships (*I_Ca_*, mean ± S.E.M.) of WT_L_ and M1354I_L_ recorded in parallel on the same days (>3-different transfections). Inset (upper): Box plots of peak current densities (median, 5/95 percentile, [pA/pF]): WT_L_: −10.9 (−72.3/-5.86) n = 38; M1354I_L_: −8.33 (−25.3/-4.44), n = 40) were significantly smaller for the mutant (Mann Whitney test, p = 0.029). Inset (lower): Representative *I_Ca_* traces of WT_L_ and M1354I_L_ upon depolarization to V_max_. (b) Current-voltage relationships (*I_Ca_*, mean ± S.E.M.) of WT_S_ and M1354I_S_ recorded in parallel on the same days (>3-different transfections). Inset (upper): peak current densities (median, 5/95 percentile, [pA/pF]): WT_S_: −7.50 (−28.5/-3.63), n = 30; M1354I_S_: −5.89 (−12.6/-3.00), n = 19) were significantly smaller for the mutant (Mann Whitney test, p = 0.037). Inset (lower): Representative *I_Ca_* traces of WT_S_ and M1354I_S_ upon depolarization to V_max_. (c) M1354I steady-state activation (circles, solid lines) and inactivation (squares, dashed lines) of WT_L_ (filled black symbols), M1354I_L_ (filled blue symbols), WT_S_ (open black symbols), and M1354I_S_ (open blue symbols). No significant changes were observed in mutant channels. Gating parameters and statistics are given in Table 3. (d) Recovery from *I_Ca_* inactivation (mean ± S.E.M.) of WT_L_ and M1345I_L_. First 300 ms are magnified in the inset. Curves were fitted using a mono-exponential function; there was no difference in the time constants (τ as mean ± S.E.M [ms]: WT_L_: 166.1 ± 20.6, n = 5; M1354I_L_: 154.5 ± 42.5, n = 5; unpaired Student’s t-test). E: Fraction of I_max_ remaining after repetitive depolarizations to V_max_ at 3 Hz (mean ± S.E.M.) of WT_L_ (n = 10) versus M1345I_L_ (n = 5). (f) Normalized inactivation time course of *I_Ca_* after 5-s depolarization to V_max_ (mean ± S.E.M.) of WT_L_ (n = 35) and M1354I_S_ (n = 9). For statistics, see Table 4.

Mutation M1354I is located in a highly conserved region at the extracellular end of segment IVS5 closely interacting with segment IIIS1 and the pore loop in domain IV [33], interactions that may be involved in coupling the voltage sensing domain to the pore domain [34]. However, in contrast to the other mutations, M1354I neither altered the voltage dependence of activation or inactivation (Figure 6(a–c), Table 3) nor the time course of inactivation during 5-s depolarizing pulses to V_max_ (Figure 6(f,g), Table 4). In addition M1354I_L_ did not change the I_tail_/Q_ON_ ratio as an indirect measure of open probability ((I_tail_/Q_ON_ [ms^−1^]: WT_L_: 15.8 ± 1.77, n = 16; M1354I_L_: 18.3 ± 1.70, n = 17, p = 0.3067, unpaired Student’s t-test). Current densities were reduced at all voltages in both long (Figure 6(a)) and short Ca_v_1.3 variants (Figure 6(b)), which could be explained by less surface expression of the mutant as evident from a significantly lower Q_ON_ as compared to WT_L_ ((Q_ON_ [pA*ms]: WT_L_: 144.7 ± 29.8, n = 16; M1354I_L_: 60.0 ± 7.38, n = 17, p = 0.008, unpaired student’s t-test).

A more detailed characterization of its gating properties further revealed no changes in recovery from inactivation determined by 10-ms test pulses to V_max_ at different time-points after a 5-s conditioning pulse to V_max_ (Figure 6(d)). Accumulation of M1354I_L_ in inactivated states induced by repetitive 3-Hz stimulation (30 100-ms steps to V_max_) did also not differ from WT (Figure 6(e)).

Taken together, our data clearly show that APA mutations F747L and R990H can lead to functional changes permitting enhanced voltage-dependent Ca^2+^ entry into APAs through different molecular mechanisms. They can therefore explain the excessive aldosterone production in these tumors. In contrast, a pathogenic role of mutation M1354I was not evident from our study.

## Discussion

We and others, have previously discovered an important pathophysiological role of gating changes of *de novo CACNA1D* mutations, which are capable of enhancing Ca^2+^ influx through Ca_v_1.3 channels. Such mutations occur as somatic mutations in APAs (resulting in treatment-resistant hypertension) and, if present germline, cause a neurodevelopmental disorder with a wide symptomatic spectrum. From a diagnostic point of view, it is important to distinguish pathogenic mutations causing the disease from other rare polymorphisms with low disease risk or no disease relevance at all.

Here we further expand our collection of functionally verified pathogenic mutations by demonstrating that APA mutation F747L exhibits very similar gating changes as previously observed for other mutations [9–12,15, Table 7], all characterized by a strong negative shift of activation and inactivation voltage. This facilitates channel opening by weaker depolarizations from negative resting membrane potentials and enables window current at subthreshold voltages. Although the time course of *I_Ca_* inactivation during long depolarizations is variable, several of the mutants (G403D, G403R, G407R) are characterized by a very strong loss of slow inactivation giving rise to a large fraction of non-inactivated current even after 5-s depolarizations to V_max_. Interestingly, in contrast to G403D and G403R [15], G407R caused no negative shift in activation voltage when introduced into the C-terminally long Ca_v_1.3 α1 splice variant [10]. However, our present data show that such a negative shift (of about −6 mV) is observed when introduced into the short splice variant. Thus, more negative activation voltage range seems to be the common hallmark of the majority of pathogenic gating-modifying *CACNA1D* mutations analyzed so far.

**Table 7.** Classification of *CACNA1D* missense mutations by characteristic functional changes.

Type	Mutation	Disease phenotype	Characteristic functional changes (channel complexes of mutant α1-subunits with α2δ1 and β3)	References
1	G403D	APAs; hyperaldosteronism or hyperinsulinism with seizures and developmental delay	**Inactivation almost abolished (voltage-dependence of inactivation not measurable)** Voltage-dependence of activation shifted to hyperpolarized voltages or unchanged	[15]
	G403R	APAs		[15]
	G407R	ASD (and ID)		[10]
2	V259D	APAs	**Voltage-dependence of activation strongly shifted to hyperpolarized voltages** Inactivation not abolished* with voltage-dependence of inactivation strongly shifted to hyperpolarized voltages or unchanged * may be faster, slower, more or less complete after 5 s depolarizations to V_max_	[11]
	V401L	ASD, ID and seizures		[9]
	F747L	APAs		[11]
	A749G	ASD (and ID)		[9,16]
	I750M	APAs; hyperaldosteronism with seizures and developmental delay		[11,15]
	V1153G	APAs		[12]
3	Q558H	ID, seizures, hearing impairment	**Slower and less complete inactivation during 3–5s depolarizations to V_max_ and** no changes in voltage-dependence of gating	[14]
	P1336R	APAs		[11]
4	R990H	APAs	**Mutation-induced (depolarizing) ω-current**	[11,21]

Splice-variant dependent gating changes are of particular interest for germline mutations, since the relative expression of Ca_v_1.3 splice variants differs across different tissues and within brain regions and might therefore result in tissue-specific phenotypes. For example, a particularly high relative expression of short splice variants is observed in dopamine neurons in the Substantia nigra [35]. Splice-variant dependent mutational effects have also been reported for other voltage-gated Ca^2+^ channels. A single missense mutation in exon 24 (III-IV cytoplasmic linker) in the α1-subunit of Ca_v_3.2 T-type channels (*CACNA1H*), which confers part of the risk for epilepsy in GAERS rats, speeds recovery from inactivation and thereby increases Ca^2+^ currents during repetitive depolarizations [36]. This phenotype was only observed in channels also containing a short exon 25. The inclusion of this exon slowed recovery from inactivation as compared to channels devoid of exon 25, and the mutation offset this effect. Likewise, alternative splicing in the C-terminal tail of Ca_v_2.1 α1-subunits of P/Q-type channels (*CACNA1A*) affected the functional changes of several gain-of-function missense mutations causing Familial Hemiplegic Migraine Type 1 [8], including S218L. It is possible that this mechanism also contributes to the differential effects of this mutation on neurotransmission at cortical pyramidal (gain-of-function of excitatory neurotransmission) and multipolar interneurons (unaltered inhibitory neurotransmission) in S218L knockin mice [37].

In addition, our data further strengthen our previous finding that ω-currents through the voltage sensor could represent an alternative molecular mechanism how *CACNA1D* mutations could drive depolarization and subsequent activation of voltage-gated Ca^2+^-entry [21]. APA mutation R990H affects the basic charge R3 in the voltage-sensor of domain III of the Ca_v_1.3 α1-subunit and allows permeation of cation flux at resting membrane potentials compatible with the formation of a water wire as predicted by molecular dynamics simulations [21]. However, to support the hypothesis of a pathogenic role of this mechanism the absence of typical gating changes as described for other mutations needs to be demonstrated. While we have shown this earlier for the long splice variant of Ca_v_1.3 (R990H_L_), we here complement our findings by also demonstrating that gain-of function gating changes are also absent in R990H_S_.

We were unable to demonstrate any functional consequences for APA mutation M1354I despite an extensive analysis of its gating properties and characterization of the mutation in both splice variants. The only significant change was a small reduction in maximal current amplitude compared to WT in both splice variants. However, based on findings in Ca_v_1.3 knockout mice and humans with *CACNA1D* mutations [19,20], a loss-of-function phenotype by a heterozygous *de novo* mutation is unlikely to be pathogenic. At present, we cannot rule out the possibility that a special combination of accessory β- and α2δ-subunits, other splice variants or a yet unknown molecular mechanism not active in our expression system can unmask functional changes in M1354I channels. An alternative possibility is that this rare variant is not responsible for excess aldosterone production in APAs. This is supported by the fact that high-confidence somatic nonsynonymous *CACNA1D* variants that passed stringent *in silico* filtering have recently been described in APCCs [22] that are also unlikely to be pathogenic. This includes two premature stop codons. One of these (R510X) must result in a truncated non-functional channel. Another APA mutation, V728I, reported previously only once in an APA of a Chinese patient [18,38] is frequently present germline in healthy individuals [39 heterozygous alleles in EXAC database]. This also questions if it alone can account for excessive aldosterone production. Since this mutation is located on the extracellular end of helix IIS6 it is outside the regions highly vulnerable to mutational gating defects [S4, S4-S5-linker, cytoplasmic end of S6; 18]. Functional characterization of this mutation must therefore address its potential pathogenic role.

Our work also has important diagnostic consequences. First, by extending the list of APA mutations for which we can demonstrate functional changes compatible with a pathogenic phenotype, we facilitate future genetic diagnosis in patients with neurodevelopmental syndromes of unknown cause. If any of these APA mutations were identified germline in such patients, this would provide robust evidence for a causal role in their disease, even if endocrine symptoms were absent. Although APA mutations can drive excess aldosterone production in these benign tumors, hyperaldosteronism has so far only been reported for two mutations in three (two with G403D, one with I750M) of the patients with germline missense mutations. No endocrine abnormalities were reported in the clinically well-studied patient carrying the V401L APA mutation [9]. At present the overall number of patients with germline mutations is too small for a reliable genotype-phenotype correlation.

Second, we show that the typical (“diagnostic”) gating changes, despite significant differences, are found in long and short Ca_v_1.3 splice variants. Therefore, for further routine diagnostics, analysis in one of the splice variants is sufficient if the diagnostic hallmarks can be demonstrated therein. Characterization of a larger set of mutations now allows us to propose four different characteristic types based on functional criteria (Table 7): *Type 1* are the inactivation-deficient mutations, in which most of the Ca_v_1.3 current fails to inactivate in Ca_v_1.3_L_. These comprise G403D, G403R, G407R. They could also be classified as “Timothy Syndrome- like” mutations because they are very similar to the Ca_v_1.2 α1-subunit (*CACNA1C*) mutations diagnostic for Timothy Syndrome [40,41]. *Type 2* mutations still inactivate to variable extents but are characterized by pronounced negative shifts of activation voltage with or without a strong negative shift also of inactivation voltage (A749G, I750M, V401L, V1153G, V259D and, as reported here, F747L). *Type 3* is characterized only by slower and less complete inactivation after 3–5 s at V_max_, which should favor persistent current in cells during prolonged depolarizations. *Type 4* are mutations in the voltage sensor (e.g. S4 helix positive charges), which enable depolarizing ω-currents as described in several other voltage-gated Ca^2+^-channels [39,42–44]. Mutations without a functional phenotype *in vitro* as shown here for M1354I should be considered rare non-pathogenic variants until further functional or clinical proof for their pathogenicity can be obtained.
